# Rückenmarknahe Regionalanästhesieverfahren im perioperativen Management bei pulmonaler Hypertonie: Eine Literaturübersicht mit 2 Fallberichten

**DOI:** 10.1007/s00101-025-01595-6

**Published:** 2025-09-30

**Authors:** Danilo Hackner, Dorothea Lange, Caroline Gräfe, Joachim Andrassy, Dionysios Koliogiannis, Patrick Scheiermann

**Affiliations:** 1 Klinik für Anaesthesiologie, LMU Klinikum München, Marchioninistr. 15, 81377 München, Deutschland; 2 Klinik für Allgemein‑, Viszeral- und Transplantationschirurgie, LMU Klinikum München, München, Deutschland

**Keywords:** Anästhesiologisches Risikomanagement, Anästhesieverfahren bei Hochrisikopatienten, Kombinierte Spinal-Epidural-Anästhesie (CSE), Anaesthesia risk management, Anaesthesia in high risk patients, Combined spinal-epidural anaesthesia (CSE)

## Abstract

**Hintergrund:**

Die pulmonale Hypertonie (PH) ist mit einer deutlichen Erhöhung der perioperativen Morbidität und Mortalität verbunden.

**Fragestellung:**

Perioperatives Management bei pulmonaler Hypertonie nach aktueller Datenlage.

**Material und Methoden:**

Analyse der aktuellen Literatur, Darstellung und Diskussion von Grundlagenarbeiten und Expertenempfehlungen sowie Präsentation zweier Fallberichte.

**Ergebnisse:**

Es besteht ein Mangel an belastbarer und qualitativ hochwertiger Literatur. Eine präzise präoperative Risikoevaluation und individuelle Therapieplanung sind bei Patient:innen mit PH obligat.

**Schlussfolgerung:**

Ein rückenmarknahes Regionalanästhesieverfahren kann bei Patient:innen mit hohem präoperativen Risikoprofil eine Alternative zur Allgemeinanästhesie sein. Zur Aufrechterhaltung physiologischer Zielparameter ist bei PH eine differenzierte Vasopressor- und Inotropikatherapie notwendig. Die frühzeitige Verlegung in ein Zentrum mit entsprechender Expertise wird bei PH empfohlen.

## Abstract (en)

**Background:**

Pulmonary hypertension (PH) is associated with a substantial increase in perioperative morbidity and mortality.

**Objective:**

Perioperative management of pulmonary hypertension according to current data.

**Material and methods:**

Analysis of the current literature, presentation and discussion of basic research and expert recommendations as well as presentation of two case reports.

**Results:**

There is a lack of reliable and high-quality literature. A precise preoperative risk evaluation and individual treatment planning are mandatory for patients with PH.

**Conclusion:**

Spinal anesthesia, thoracic epidural anesthesia or a combination of both can be an alternative to general anesthesia in patients with a high preoperative risk profile. A differentiated vasopressor and inotropic treatment is necessary to maintain physiological target parameters in PH. The early referral to a center with appropriate expertise is recommended in PH.

Die pulmonale Hypertonie (PH) und damit verbundene Einschränkungen der rechtskardialen Pumpfunktion sind verbunden mit einer deutlichen Erhöhung der perioperativen Morbidität und Mortalität. Das perioperative Management umfasst neben der intraoperativen Betreuung auch die präoperative Risikoevaluation, die medikamentöse Therapieoptimierung und die Planung des Prozedere sowie die postoperative Überwachung. Jeder dieser Aspekte erfordert ein fundiertes Wissen, um Komplikationen frühzeitig zu antizipieren und so eine Dekompensation der Erkrankung zu verhindern. Am Ende des Artikels veranschaulichen zwei ausgewählte Patientenfälle eine mögliche Umsetzung rückenmarknaher Regionalanästhesieverfahren in der klinischen Praxis.

## PH – eine Übersicht

Durch die diversen Ursachen wird mit dem Begriff der PH noch keine Diagnose gestellt, sondern es werden die hämodynamischen Veränderungen im Lungenkreislauf beschrieben; die Inzidenz und Prävalenz variiert erheblich zwischen den verschiedenen Entitäten [1]. Es wird von einer Prävalenz von etwa 1 % der Weltbevölkerung ausgegangen, wobei diese bei über 65-Jährigen auf bis zu 10 % steigen kann [2] und Frauen insbesondere bei der pulmonalarteriellen Hypertonie (PAH; WHO[World Health Organization]-Gruppe I) überrepräsentiert sind. Die PH ist definiert durch einen mittleren pulmonalen arteriellen Druck („mean pulmonary arterial pressure“, PAPm) von mehr als 20 mm Hg in Ruhe (Norm: 12–16 mm Hg). Häufigste Ursachen sind Linksherzerkrankungen und vaskuläre Veränderungen der Lunge. Die gemeinsame Endstrecke aller Krankheitsentitäten liegt in der Rechtsherzbelastung bis hin zum Rechtsherzversagen. Unbehandelt beträgt die mediane Überlebenszeit bei PH etwa 2 bis 3 Jahre ab Diagnosestellung [3]. Durch moderne Therapiekonzepte konnte die 5‑Jahres-Überlebensrate auf etwa 57 % verbessert werden [4]. Patienten, die auf Kalziumkanalblocker ansprechen (ca. 10 %), zeigen eine besonders günstige Prognose mit einer 5‑Jahres-Überlebensrate von bis zu 87 % [5]. Der Verlauf ist meist progredient, wobei zunehmende Belastungsdyspnoe und Zeichen des Rechtsherzversagens im Vordergrund stehen. Viele Patienten werden erst in einem fortgeschrittenen Krankheitsstadium diagnostiziert, was die Prognose erheblich verschlechtert. Zudem unterscheidet sich die Prognose je nach zugrunde liegender Pathologie deutlich. Patienten mit PH infolge einer Linksherzerkrankung oder einer chronischen Lungenerkrankung weisen tendenziell schlechtere Überlebensraten auf [6].

### Klassifikation und Pathogenese

**Abb. 1:**
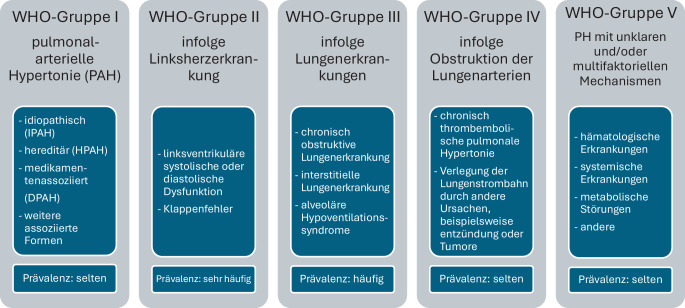
Klassifikation der pulmonalen Hypertonie (*PH*) der European Society of Cardiology (ESC) und der European Respiratory Society (ERS; modifiziert nach [1, 2]). *WHO* World Health Organization

Gemeinsamkeiten in Pathogenese, klinischer Manifestation, hämodynamischen Merkmalen und Therapie bedingen die aktuelle Klassifikation der PH der European Society of Cardiology (ESC) und der European Respiratory Society (ERS) von 2022 [7]. Hierbei wird die PH klinisch in 5 Gruppen (Abb. 1) unterteilt. Die seltene Form der WHO-Gruppe I hat eine Prävalenz von etwa 15 bis 50 Fällen pro 1 Mio. Einwohner, wohingegen die WHO-Gruppe II die häufigste Form darstellt und bei bis zu 60 % der Patienten mit systolischer oder diastolischer Herzinsuffizienz auftritt.

Dieser klinischen Einteilung steht eine weitere – die Einteilung nach Hämodynamik – gegenüber. Hierfür ist neben dem PAPm die Kenntnis weiterer kardialer Drücke notwendig, welche die invasive Messung der Hämodynamik mittels Rechtsherzkatheter (RHK) als Goldstandard erforderlich machen. Neben dem pulmonalarteriellen Verschlussdruck, auch Wedge-Druck genannt („pulmonary artery wedge pressure“ [PAWP], Norm: ≤ 15 mm Hg), welcher die Abschätzung des linksventrikulären enddiastolischen Drucks erlaubt, wird der pulmonale vaskuläre Widerstand („pulmonary vascular resistance“ [PVR], Norm: < 3 Wood-Einheiten [WE]) benötigt. So ist die Unterscheidung zwischen einer primär pulmonalen Ursache oder auch einer präkapillären PH (WHO Gruppe I, III, IV) und einer postkapillären PH, der eine Linksherzerkrankung zugrunde liegt (WHO-Gruppe II), möglich. Auch die Abgrenzung von Mischformen (kombinierte prä- und postkapilläre PH) und der unklassifizierten PH (WHO-Gruppe V) kann so erfolgen. Tab. 1 fasst die entsprechenden hämodynamischen Merkmale der verschiedenen Definitionen zusammen. Der Vollständigkeit halber ist hier auch die Definition der Belastungs-PH aufgeführt, welche in der aktuellen Überarbeitung der Leitlinie wieder eingeführt wurde, da so eine Früherkennung von Lungengefäßerkrankungen in einem frühen Stadium möglich ist [8].

**Tab. 1:** Hämodynamische Definition der pulmonalen Hypertonie (modifiziert nach [7, 8])

Definition	mPAP	PAWP	PVR	Hämodynamische Merkmale
PH	↑	–	–	mPAP > 20 mm Hg
Präkapilläre PH	↑	↔	↑	mPAP > 20 mm Hg PAWP ≤ 15 mm Hg PVR > 2WE
Isolierte postkapilläre PH (IpcPH)	↑	↑	↔	mPAP > 20 mm Hg PAWP > 15 mm Hg PVR ≤ 2 WE
Kombinierte prä- und postkapilläre PH (CpcPH)	↑	↑	↑	mPAP > 20 mm Hg PAWP > 15 mm Hg PVR > 2WE
Unklassifizierte PH	↑	↔	↔	mPAP > 20 mm Hg PAWP ≤ 15 mm Hg PVR ≤ 2 WE
Belastungs-PH	–	–	–	mPAP/CO-Steigung zwischen Ruhe und Belastung > 3 mm Hg/l/min

↑ erhöht, ↔ normwertig, *mPAP* mittlerer pulmonalarterieller Druck („mean pulmonary arterial pressure“), *PAWP* pulmonalarterieller Verschlussdruck („pulmonary artery wedge pressure“), *PVR* pulmonal vaskulärer Widerstand („pulmonary vascular resistance“), *WE* Wood-Einheiten, *CO* Herzminutenvolumen

### Symptome und Diagnostik

**Abb. 2:**
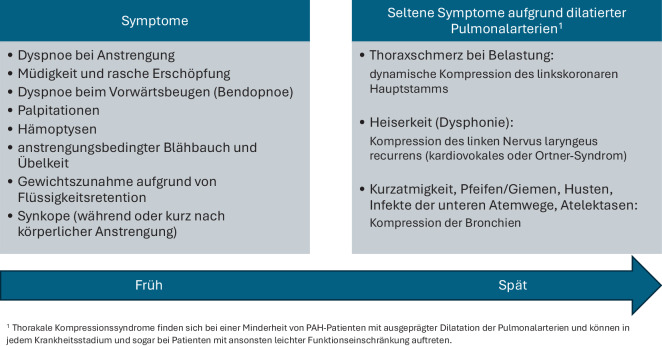
Symptome bei pulmonaler Hypertonie (modifiziert nach [7]). *PAH* pulmonalarterielle Hypertonie

Geprägt durch die Dysfunktion des rechten Ventrikels, äußert sich die PH initial meist in einer progredienten Belastungsdyspnoe. Typischerweise zeigen sich in frühen Krankheitsstadien geringe bis milde Symptome, und es kommt erst im weiteren Krankheitsverlauf zu einer Beschwerdezunahme, v. a. unter Belastung, und weiteren – meist unspezifischen – Symptomen. Abb. 2 gibt einen Überblick über häufige Symptome sowohl im frühen als auch im fortgeschrittenen Krankheitsverlauf. Besonderes Augenmerk sollte v. a. in der Initialphase auf eine mögliche PH-induzierende Grunderkrankung gelegt werden, deren Symptome lange im Vordergrund stehen können. Im Spätstadium zeigen sich neben Symptomen aufgrund dilatierter Pulmonalarterien (siehe Abb. 2) auch Zeichen der Rechtsherzdekompensation wie Ödeme, Aszites und Halsvenenstauung.

Neben der initialen Diagnostik, bestehend aus körperlicher Untersuchung (siehe Tab. 2), EKG, Röntgenthorax, Laborwerten und Lungenfunktionsuntersuchungen, hat v. a. die transthorakale Echokardiographie (TTE) einen hohen Stellenwert und kann bei Zeichen einer Rechtsherzbelastung wegweisend für eine mögliche PH sein. Aufgrund der komplexen Geometrie des rechten Ventrikels und der heterogenen Natur der PH erlaubt jedoch kein echokardiographischer Parameter allein eine zuverlässige Beurteilung des PH-Status und der zugrunde liegenden Ätiologie [7].

**Tab. 2:** Diagnostische Merkmale bei pulmonaler Hypertonie (*PH*)

Zeichen für PH	Zentrale, periphere oder gemischte Zyanose
	Akzentuierte pulmonale Komponente des zweiten Herztons
	RV dritter Herzton
	Systolisches Herzgeräusch der Trikuspidalklappeninsuffizienz
	Diastolisches Herzgeräusch bei Pulmonalklappeninsuffizienz
Zeichen, die auf eine zugrunde liegende Ursache der PH hinweisen	Trommelschlägelfinger: zyanotische CHD, fibrotische Lungenerkrankung, Bronchiektasie, PVOD oder Lebererkrankung
	Differenzialdiagnose Trommelschlägelfinger/Zyanose: PDA/Eisenmenger-Syndrom
	Auskultatorische Befunde (Rasselgeräusche oder Stridor, Herzgeräusche): Lungen- oder Herzerkrankung
	Folgeerscheinungen einer TVT, Veneninsuffizienz: CTEPH
	Teleangiektasie: HHT oder SSc
	Sklerodaktylie, Raynaud-Phänomen, digitale Ulzeration, GERD: SSc
Zeichen eines Rückwärtsversagens des rechten Ventrikels	Gestaute und pulsierende Jugularvenen
	Abdominaler Blähbauch
	Hepatomegalie
	Aszites
	Periphere Ödeme
Zeichen eines Vorwärtsversagens des rechten Ventrikels	Periphere Zyanose (blaue Lippen und Akren)
	Schwindel
	Blässe
	Kühle Extremitäten
	Verlängerte kapilläre Füllungszeit

*CHD* angeborener Herzfehler („congenital heart diusease“), *CTEPH* chronisch thromboembolische pulmonale Hypertonie, *GERD* gastroösophageale Refluxerkrankung („gastroesophageal reflux disease“), *HHT* hereditäre hämorrhagische Teleangiektasie, *PDA* persitierender (offener) Ductus arteriosus, *PVOD* pulmonale venookklusive Erkrankung („pulmonary veno-occlusive disease“), *RV* rechtsventrikulär/rechter Ventrikel, *SSc* systemische Sklerose, *TV* tiefe Venenthrombose

Weitere Diagnostik umfasst neben dem RHK auch eine High-Resolution-Computertomographie (CT) zur Detektion einer möglicherweise zugrunde liegenden Lungenparenchymerkrankung, eine Perfusions- und Ventilationsszintigraphie der Lunge sowie eine genetische Analyse bei Verdacht auf eine hereditäre Form der PH.

## Implikationen im perioperativen Kontext

Der Mangel an belastbarer, qualitativ hochwertiger Evidenz führt dazu, dass nach wie vor keine formale Leitlinie zum Management dieser vulnerablen Patientengruppe vorliegt. Auf Basis von Expertenmeinungen gibt es sowohl nationale als auch internationale Handlungsempfehlungen, so z. B. die Empfehlung der Kölner Konsensus-Konferenz von 2018 [9] oder das Consensus Statement der International Society for Heart and Lung Transplantation (ISHLT) von 2022 [10].

Die Gründe für die erhöhte perioperative Morbidität und Mortalität von Patienten mit PH sind vielfältig, und das Risiko ist neben dem Grad der PH-Ausprägung auch abhängig von Art und Dringlichkeit des operativen Eingriffs. Insbesondere Notfalleingriffe scheinen mit besonders hoher Morbidität und Mortalität assoziiert zu sein [11, 12].

Die Rechtsherzinsuffizienz ist die häufigste Todesursache bei diesen Patienten. Während der Anästhesie führen Faktoren wie Hypoxie, Hyperkapnie, Azidose, Hypothermie und Volumenüberladung zu einer weiteren Erhöhung des pulmonalen Widerstands und damit zur akuten Dekompensation. Wesentliche anästhesiologische Gefahren umfassen:

- akute Rechtsherzdekompensation durch Erhöhung der Nachlast,
- Hypotonie bei verminderter rechtsventrikulärer (RV) Auswurfleistung,
- Hypoxämie durch gestörte pulmonale Perfusion und Diffusionsprobleme,
- Arrhythmien infolge myokardialer Ischämie und Dehnung.

Eine detaillierte präoperative Risikoevaluation und eine individuelle Therapieplanung sollten deshalb – wann immer möglich – durchgeführt werden [13–15]. Die präoperative Risikoevaluation sollte hier neben Anamnese und körperlicher Untersuchung auch apparative Untersuchungen beinhalten [9]. Hierzu kann auch eine Orientierung am Drei-Strata-Modell der ESC/ERS zur Risikostratifizierung bei Diagnosestellung erfolgen (siehe Tab. 3). Für die präoperative Evaluation sind besonders folgende Kenntnisse der Grunderkrankung wichtig:

- Ätiologie und Klassifikation der PH:
  - Jede Gruppe der PH unterscheidet sich in Prognose, Reversibilität und therapeutischen Möglichkeiten.
- Schweregrad und hämodynamisches Profil:
  - Eine PH mit hohem pulmonalvaskulären Widerstand und niedriger Herzleistung ist mit einem besonders hohen perioperativen Risiko verbunden.
- Rechtsherzfunktion und Kompensation:
  - Bestehen eine kompensierte Rechtsherzfunktion oder bereits Zeichen des Rechtsherzversagens? Besonderes Augenmerk sollte auf das Erkennen latenter oder manifester Dekompensationszeichen gelegt werden.
- Symptomatische Belastbarkeit:
  - Patienten mit eingeschränkter Belastbarkeit (WHO-Funktionsklasse III oder IV) haben ein deutlich erhöhtes perioperatives Risiko.
- Aktuelle medikamentöse Therapie:
  - Fortführen bzw. Optimierung der spezifischen PH-Medikation ist elementar, plötzliche Unterbrechungen der Medikation können zu einer raschen klinischen Verschlechterung führen
- Begleitpathologien:
  - Häufig bestehen relevante Komorbiditäten wie chronische Hypoxie, Obstruktion der Atemwege, koronare Herzkrankheit oder Koagulopathien, welche in die Risikoabwägung und präoperative Optimierung einfließen.

**Tab. 3:** Drei-Strata-Modell der European Society of Cardiology (ESC) und der European Respiratory Society (ERS) (nach [7])

Prognoseparameter (geschätzte 1‑Jahres-Sterblichkeit)	Niedriges Risiko (< 5 %)	Intermediäres Risiko (5–20 %)	Hohes Risiko (> 20 %)
Klinische Zeichen und modifizierbare Parameter			
Zeichen einer Rechtsherzinsuffizienz	Nicht vorliegend	Nicht vorliegend	Vorliegend
Progression der Symptome und der klinischen Manifestationen	Nein	Langsam	Schnell
Synkope	Nein	Gelegentliche Synkopen^a^	Wiederholte Synkopen^b^
WHO-FC	I, II	III	IV
6MWD^c^	> 440 m	165–440 m	< 165 m
CPET	peakVO_2_ > 15 ml/min/kg (> 65 % Soll)	peakVO_2_ 11–15 ml/min/kg (35–65 % Soll)	peakVO_2_ < 11 ml/min/kg (< 35 % Soll)
	VE/VCO_2_-Slope < 36	VE/VCO_2_-Slope 36–44	VE/VCO_2_-Slope > 44
Biomarker: BNP oder NT-proBNP^d^	BNP < 50 ng/l	BNP 50–800 ng/l	BNP > 800 ng/l
	NT-proBNP < 300 ng/l	NT-proBNP 300–1100 ng/l	NT-proBNP > 1100 ng/l
Echokardiographie	RA-Fläche < 18 cm^2^	RA-Fläche 18–26 cm^2^	RA-Fläche > 26 cm^2^
	TAPSE/PAPs > 0,32 mm/mm Hg	TAPSE/PAPs 0,19–0,32 mm/mm Hg	TAPSE/PAPs < 0,19 mm/mm Hg
	Kein Perikarderguss	Minimaler Perikarderguss	Mäßiger oder großer Perikarderguss
CMR^e^	RVEF > 54 %	RVEF 37–54 %	RVEF < 37 %
	SVI > 40 ml/m^2^	SVI 26–40 ml/m^2^	SVI < 26 ml/m^2^
	RVESVI < 42 ml/m^2^	RVESVI 42–54 ml/m^2^	RVESVI > 54 ml/m^2^
Hämodynamik	RAP < 8 mm Hg	RAP 8–14 mm Hg	RAP > 14 mm Hg
	CI ≥ 2,5 l/min/m^2^	CI 2,0–2,4 l/min/m^2^	CI < 2,0 l/min/m^2^
	SVI > 38 ml/m^2^	SVI 31–38 ml/m^2^	SVI < 31 ml/m^2^
	SvO_2_ > 65 %	SvO_2_ 60–65 %	SvO_2_ < 60 %

*6MWD* 6-Minuten-Gehstrecke („6-minute walk distance“), *BNP* „brain natriuretic peptide“, *CI* Herzindex, *CMR* kardiale Magnetresonanztomographie, *CPET* Spiroergometrie („cardiopulmonary exercise testing“), *NT-proBNP* „N-terminal pro brain natriuretic peptide“, *PAH* pulmonalarterielle Hypertonie, *RA* rechter Vorhof, *RAP* rechtsatrialer Druck, *PAPs* systolischer pulmonal arterieller Druck („systolic pulmonary arterial pressure“), *SvO*_*2*_ gemischt-venöse Sauerstoffsättigung, *RVESVI* rechtsventrikulärer endsystolischer Volumenindex, *RVEF* rechtsventrikuläre Ejektionsfraktion, *Soll* Sollwert, *SVI* Schlagvolumenindex, *TAPSE* „tricuspid annular plane systolic excursion“, *VE/VCO*_*2*_ Atemäquivalent für Kohlendioxid, *VO*_*2*_ Sauerstoffaufnahme, *WHO* World Health Organization, *FC* Funktionsklasse

^a^ Gelegentliche Synkope bei schwerer körperlicher Anstrengung oder gelegentliche orthostatische Synkope bei einem stabilen Patienten

^b^ Wiederholte Episoden von Synkopen auch bei geringer oder regelmäßiger körperlicher Aktivität

^c^ Beachten Sie, dass die 6MWD von Alter, Körpergröße und Belastung durch Begleiterkrankungen abhängt.

^d^ Zur Harmonisierung mit dem in Tab. 18 in der Langfassung der ESC/ERS-Leitlinie dargestellten Vier-Strata-Modell wurden die BNP- und NT-proBNP-Cut-off-Werte gegenüber der Version von 2015 auf der Grundlage der Daten des REVEAL-Registers aktualisiert, wobei anerkannt wurde, dass die europäischen Validierungsstudien die ursprünglichen Cut-off-Werte verwendet haben.

^e^ CMR-Parameter, angepasst an Abschnitt 6.2.2.2 der Langfassung der ESC/ERS-Leitlinie

Das perioperative Risiko bei PH ist variabel und hängt nicht allein von der Grunderkrankung ab. Entscheidend sind der Schweregrad der PH sowie die Komplexität des chirurgischen und anästhesiologischen Vorgehens. Nur die integrative Betrachtung ermöglicht eine präzise Risikostratifizierung. So ist auch nicht bei allen PH-Patienten ein RHK erforderlich. V. a. in der präoperativen Planung sollte dieser dann in Betracht gezogen werden, wenn klinische Hinweise auf eine schwere PH oder RV-Dysfunktion vorliegen und die Ergebnisse voraussichtlich therapeutische Konsequenzen haben [3]. Die frühzeitige Verlegung in ein Zentrum mit entsprechender Expertise wird ausdrücklich empfohlen. Ist dies, z. B. in einer Notfallsituation, nicht möglich, sollte zumindest die konsiliarische Kontaktaufnahme mit einem entsprechenden Zentrum zur Besprechung einer optimalen Behandlungsstrategie in Erwägung gezogen werden [10].

## Präoperative Maßnahmen

Eine präoperative Optimierung der medikamentösen Therapie der PH und somit der Hämodynamik ist anzustreben, wann immer es die Dringlichkeit des chirurgischen Eingriffs zulässt. In Abhängigkeit von der Grunderkrankung kommen hierfür unter anderem Medikamente zur Senkung des pulmonalarteriellen Drucks (z. B. PDE[Phosphodiesterase]-5-Inhibitoren, Endothelinrezeptorantagonisten, sGC[„soluble guanylate cyclase“]-Stimulatoren, Prostazyklinanaloga oder Prostazyklinrezeptoragonisten) in Betracht [1].

Zusätzlicher Fokus sollte auf die Blutgerinnung gelegt werden. Aufgrund des progredienten Rechtsherzversagen kann es zu einer Stauungsleber mit Einschränkung der Blutgerinnung und konsekutiv erhöhtem Risiko für perioperative Blutungskomplikationen kommen. Die präoperative Erfassung der Leberfunktion und der Gerinnungsparameter ist daher essenziell für das Risikomanagement.

## Intraoperatives anästhesiologisches Vorgehen

In der aktuellen Literatur finden sich keine Studien, die eindeutig ein Regionalverfahren gegenüber einer Allgemeinanästhesie bevorzugen. Nichtsdestotrotz wird von Experten im elektiven Kontext, insbesondere bei schweren Formen der PH, die Durchführung eines Regionalverfahrens empfohlen [10].

Gründe für das erhöhte Risiko einer Allgemeinanästhesie sind neben dem Einfluss der verabreichten Medikamente auf die Hämodynamik insbesondere die veränderten intrathorakalen und transpulmonalen Druckverhältnisse während der maschinellen Beatmung [16]. Sowohl der positive Inspirationsdruck als auch der positive endexspiratorische Druck („positive end-expiratory pressure“, PEEP) führen über die Behinderung des venösen Rückstroms zu einer Reduktion der RV-Vorlast. Hohe Tidalvolumina und hohe PEEP-Drücke erhöhen den PVR und damit die RV-Nachlast durch Kompression kleiner pulmonaler Kapillaren, während niedrige Tidalvolumina und PEEP-Drücke zur Atelektasenbildung führen, welche über die hypoxisch-pulmonale Vasokonstriktion (HPV) ebenfalls in einer Erhöhung des PVR resultiert [10]. Auch Einschränkungen des Gasaustausches in Form von Hypoxie oder Hyperkapnie führen über die HPV zur Erhöhung des PVR.

Sollte eine Allgemeinanästhesie unumgänglich sein, empfiehlt es sich, Oxygenierung und Decarboxylierung engmaschig mittels arterieller Blutgasanalysen zu überwachen. Der PEEP sollte eher niedrig gewählt werden (5–10 mbar), die Ermittlung des „optimal PEEP“ mittels einschlägiger PEEP-Trials ist sinnvoll [17]. Das Atemminutenvolumen sollte so gewählt werden, dass pH und pCO_2_ im Normbereich liegen [18–20]. Eine gute Übersicht über das Vorgehen bei Allgemeinanästhesie und Sedierung kann der Publikation von Price et al. entnommen werden [21].

Bei der Anwendung von rückenmarknahen Regionalverfahren kann der oben genannte negative Effekt der invasiven Beatmung vermieden werden, dennoch bestehen auch hier Risiken. Bei Spinalanästhesie sowie Periduralanästhesie kommt es durch die Blockade der sympathischen Fasern zu einer peripheren Vasodilatation und damit einhergehend zu einem Abfall der RV-Vorlast und des systemisch-vaskulären Widerstands („systemic vascular resistance“ [SVR]) [22]. Insbesondere bei der thorakalen Epiduralanästhesie sind bedeutsame hämodynamische Effekte möglich, da sie neben der thorakalen auch zu einer lumbalen Sympathikusblockade führt, wenngleich Letztere sich langsamer einstellt als bei einer Spinalanästhesie [23]. In jedem Fall sollte der zu erwartenden Hypotonie beim Einbringen des Lokalanästhetikums mit einem frühzeitigen Einsatz von Vasopressoren begegnet werden.

Weiterhin ist bei in Regionalanästhesie durchgeführten Eingriffen besondere Vorsicht hinsichtlich einer begleitenden Analgosedierung geboten. Der deletäre Effekt einer Hypoxie und/oder Hyperkapnie, verursacht durch eine (zu) tiefe Analgosedierung, darf nicht unterschätzt werden und macht auch hier eine engmaschige Überwachung der arteriellen Sauerstoff- und Kohlendioxidpartialdrücke notwendig [10].

Laparoskopische Verfahren sollten bei PH zugunsten eines offenen Vorgehens verlassen werden [22]. Neben dem Kapnoperitoneum beeinflussen die bei laparoskopischen Eingriffen oft erforderlichen Lagerungsmanöver den venösen Rückstrom und damit die RV-Vorlast in nur schwer kontrollierbarem Ausmaß und erhöhen so das Risiko einer akuten RV-Dekompensation [24–27].

Euvolämie dient der Aufrechterhaltung der RV-Vorlast unter Vermeidung einer Volumenüberladung, Hypo- und Hypervolämien sollten vermieden werden. Normoxie, Normokapnie und Normothermie helfen, eine HPV und damit ein Ventilations-Perfusions-Mismatch zu verhindern. Ein adäquater arterieller Mitteldruck und die Aufrechterhaltung des SVR gewährleisten eine ausreichende Koronarperfusion sowie damit die Kontraktilität des Myokards und einen angemessenen kardialen Auswurf.

Zur intraoperativen Überwachung der Hämodynamik ist ein entsprechendes intraoperatives Monitoring notwendig. Dies umfasst neben EKG und Sauerstoffsättigung zumindest einen arteriellen und einen zentralvenösen Katheter [10]. Weiterhin kommen, je nach Schwere der PH, Art der Anästhesie und Invasivität des Eingriffs, auch ein pulmonalarterieller Katheter (PAK) und eine transösophageale Echokardiographie (TEE) in Betracht. Zu beachten ist, dass keine Evidenz für den perioperativen Einsatz des PAK bei nicht herzchirurgischen Eingriffen vorliegt. Daher sollten die Vorteile einer erweiterten hämodynamischen Überwachung und die möglichen Risiken einer PAK-Anlage sorgfältig gegeneinander abgewogen werden [28].

Zum Erreichen der oben genannten hämodynamischen Zielparameter ist eine differenzierte Inotropika- und Vasopressortherapie notwendig. Bis heute existieren zu wenige verlässliche Daten, die eine evidenzbasierte Empfehlung eines oder mehrerer Medikamente erlauben [29]. Im klinischen Alltag hat sich v. a. Norepinephrin etabliert [19, 30], Vasopressin kommt als Alternative in Betracht [31, 32]. Epinephrin und Dobutamin steigern die kardiale Kontraktilität und damit das Herzzeitvolumen, erhöhen aber den myokardialen Sauerstoffverbrauch und können so myokardiale Ischämien verursachen [33]. Insgesamt werden (Tachy‑)Arrhythmien bei PH schlecht toleriert, was den Einsatz von Epinephrin und Dobutamin limitiert [10]. Intravenös appliziertes Milrinon führt ebenfalls zu einer Steigerung der myokardialen Kontraktilität und zu einer erwünschten Senkung des PVR, jedoch auf Kosten eines gleichzeitigen Abfalls des SVR, und ist daher ebenfalls nur mit entsprechender Vorsicht bzw. unter gleichzeitiger Gabe eines Vasopressors einzusetzen [10]. Levosimendan kann eine Alternative darstellen, da es einerseits die myokardiale Kontraktilität verbessert, ohne den Sauerstoffverbrauch wesentlich zu erhöhen, und andererseits eine milde Vasodilatation im Lungenkreislauf induziert, wodurch der PVR und damit die RV-Nachlast sinkt. Levosimendan hat einen Stellenwert, wenn unter Dobutamin nur eine unzureichende hämodynamische Stabilisierung erreicht wird oder wenn das arrhythmogene Risiko von Dobutamin als zu hoch eingeschätzt wird. Allerdings ist der Einsatz von Levosimendan aufgrund des verzögerten Wirkeintritts (Wirkmaximum erst nach mehreren Stunden trotz intravenöser Gabe) in akuten Situationen begrenzt. Grundsätzlich sollte die Therapie immer in Kombination mit einer sorgfältigen Volumensteuerung erfolgen, da sowohl Hypo- wie auch Hypervolämie für den rechten Ventrikel kritisch sind.

Die inhalative Gabe von Milrinon, Prostazyklinanaloga oder NO zur RV-Nachlast-Senkung ist prinzipiell intraoperativ möglich, es gibt jedoch keine ausreichende Evidenz, welche den routinemäßigen Einsatz stützt. Im Falle einer hämodynamischen Dekompensation, wenn der Einsatz von intravenösen Inotropika und Vasopressoren nicht ausreichend wirksam ist, kann dies in Erwägung gezogen werden [10].

## Postoperative Betreuung

Da die meisten postoperativen Komplikationen innerhalb der ersten 48–72 h auftreten, ist die Indikation für eine Überwachung auf einer Intensivstation über diesen Zeitraum gegeben.

Das größte Risiko besteht für eine akute rechtskardiale Dekompensation. Als auslösende Faktoren kommen neben einer postoperativen respiratorischen Insuffizienz mit Hypoxie und Hyperkapnie auch Hypotonien, Volumenverschiebungen, (Tachy‑)Arrhythmien und Infektionen in Betracht [10]. Zum Assessment der kardialen Pumpfunktion kommt die TTE oder auch die TEE infrage. Eine diastolische Funktionsstörung des RV birgt das Risiko einer Dekompensation sowohl bei Reduktion als auch bei Erhöhung der RV-Vorlast, sodass beide Extreme vermieden werden müssen. Auch wenn der zentrale Venendruck (ZVD) nicht zwangsläufig mit der RV-Vorlast korreliert, können sich die kontinuierliche ZVD-Messung und die Beobachtung des Trends als hilfreich erweisen [10]. Test auf Volumenreagibilität, wie z. B. die Mini-Fluid-Challenge oder der Passive-Leg-Raise-Test geben einen Anhalt, ob eine weitere Flüssigkeitsgabe gerechtfertigt ist [34]. Andererseits signalisieren ein erhöhter ZVD (> 15 mm Hg; [10]) oder sonographische Zeichen einer venösen Stauung (z. B. erhöhter VExUS[„venous excess ultrasound“]-Score; [35]) die Notwendigkeit, eine weitere Flüssigkeitszufuhr zu unterbinden bzw. eine Negativbilanzierung anzustreben.

Zur Vermeidung einer postoperativen respiratorischen Insuffizienz sollten frühzeitig atem- und physiotherapeutische Maßnahmen zur Atelektaseprophylaxe zur Anwendung kommen. Zur Vermeidung der HPV sollte ein Ziel-SpO_2_ (Sauerstoffpartialdruck) von mehr als 92 % angestrebt werden, die Gabe von Sauerstoff sollte hierbei liberal erfolgen. Ebenso sollten die hoch dosierte Gabe von Sauerstoff mittels HFNC („high-flow nasal cannula“) oder die nicht-invasive Ventilation frühzeitig eingesetzt werden, wenn dadurch eine (Re‑)Intubation vermieden werden kann. Der Einsatz inhalativer Vasodilatoren kann zudem helfen, ein Ventilations-Perfusions-Missverhältnis zu verbessern [10].

Eine adäquate Schmerztherapie ist notwendig, da Schmerzen mit einer Erhöhung des Sauerstoffbedarfs und des PVR einhergehen. Da der Einsatz von Opioiden mit der Gefahr von Hypoventilation assoziiert ist, sollten vor deren Anwendung primär peripher wirkende Schmerzmedikamente sowie kathetergestützte Regionalanästhesieverfahren zum Einsatz kommen [9, 15].

## Fazit für die Praxis

- Eine Kombination aus Spinalanästhesie und thorakaler Periduralanästhesie kann bei Patient:innen mit hohem präoperativen Risikoprofil eine Alternative zur Allgemeinanästhesie sein.
- Eine präzise präoperative Risikoevaluation und eine individuelle Therapieplanung sind bei Patient:innen mit PH obligat.
- Eine präoperative Optimierung der medikamentösen Therapie bei PH ist anzustreben, wann immer es die Dringlichkeit des Eingriffs zulässt.
- Zur Aufrechterhaltung physiologischer Zielparameter ist bei PH eine differenzierte Vasopressor- und Inotropikatherapie notwendig.
- Die frühzeitige Verlegung in ein Zentrum mit entsprechender Expertise wird bei PH empfohlen.

## Anhang

### Patientenfälle

#### Fallbericht I: Sigmaresektion bei schwerer PH

Wir berichten über einen 49-jährigen Patienten mit schwerem pulmonalen Hypertonus als Folge einer Sarkoidose. Es bestand eine hypoxische respiratorische Insuffizienz (respiratorische Insuffizienz Typ I), welche eine Langzeitsauerstofftherapie (Dosis 2–3 l/min) notwendig machte. Das chronische Cor pulmonale hatte bereits in der Vorgeschichte zu rezidivierenden kardialen Dekompensationen geführt.

Im Rahmen der Evaluation zur Listung für eine Lungentransplantation wurde bei einer Koloskopie ein über die Länge von ca. 6 cm zirkulär wachsendes Karzinom im distalen Sigma diagnostiziert. Es wurde die Indikation zur zeitnahen operativen Therapie gestellt, wobei man sich aufgrund der kardiopulmonalen Komorbiditäten auf ein offenes Operationsverfahren statt des zunächst geplanten laparoskopischen Vorgehens einigte.

Der Patient wurde im Rahmen der anästhesiologischen Prämedikationsvisite evaluiert. In der echokardiographischen Untersuchung imponierte ein deutlich dilatiertes und hypertrophiertes rechtes Herz (rechtes Atrium [RA]: planimetrisch 38 cm^2^, enddiastolischer RV-Durchmesser [RVEDD]: 6,9 cm) mit reduzierter Pumpfunktion (TAPSE: 13 mm) und hochgradiger Trikuspidalklappeninsuffizienz. Der linke Ventrikel wurde durch den dilatierten rechten Ventrikel komprimiert (sog. „D sign“, linksventrikulärer enddiastolischer Durchmesser [LVEDD]: 2,3 cm), die linksventrikuläre Ejektionsfraktion (LVEF) war erhalten.

In der Lungenfunktionsprüfung (LuFu) zeigte sich das Bild einer schweren Restriktion ohne nennenswerte Obstruktion (VC_max_ [maximale Vitalkapazität]: 1,77 l, FEV_1_ [Einsekundenkapazität]: 1,36 l) sowie eine hochgradige Diffusionsstörung (DLCOc/VA [eingeschränkter Transferfaktor als Ausdruck des Verhältnisses zwischen der Diffusionskapazität für Kohlenmonoxid (DLCO) und der alveolären Ventilation (VA)] 0,15 mmol/min/kPa/l, entsprechend 10 % des Sollwerts). Eine kapilläre Blutgasanalyse unter Raumluftbedingungen bestätigte die respiratorische Insuffizienz Typ I (pO_2_: 38,3 mm Hg, pCO_2_: 40,2 mm Hg). In einer RHK 3 Monate vor der geplanten Operation war ein PAPm von 70 mm Hg sowie ein PAWP von 12 mm Hg gemessen worden. Herzzeitvolumen bzw. Herzindex waren zu diesem Zeitpunkt mit 3,4 l/min respektive 1,8 l/min/m^2^ deutlich reduziert.

Nach ausführlicher Diskussion fiel die Wahl des Anästhesieverfahrens auf ein kombiniertes neuraxiales Verfahren in Form einer thorakalen Periduralanästhesie (PDA) sowie einer separaten Spinalanästhesie (SPA) mit begleitender Analgosedierung.

Neben einer arteriellen Kanüle (A. radialis links) zur invasiven Blutdruckmessung und einem 4‑Lumen-ZVK (zentraler Venenkatheter; V. jugularis interna rechts) zur Applikation von Medikamenten wurden noch 2 venöse (V. jugularis interna rechts [8,5 Fr] und V. femoralis rechts [6 Fr]) und eine arterielle Einführschleuse (A. femoralis rechts [4 Fr]) in Lokalanästhesie gelegt. Diese Einführbestecke sollten das Einschwemmen eines PAK bzw. die Anlage einer venoarteriellen extrakorporalen Membranoxygenierung (VA-ECMO) im Notfall ermöglichen.

Die Anlage der thorakalen PDA auf Höhe Th 9/10 gelang technisch problemlos, ebenso die folgende Spinalanästhesie auf Höhe L 2/3. Bei prompt einsetzender Sympathikusblockade waren jedoch bereits zu diesem Zeitpunkt eine inotrope Unterstützung mittels niedrig dosiertem Epinephrin (Dosis: 0,04–0,07 µg/kg Körpergewicht [KG]/min) sowie die Gabe von Norepinephrin (Dosis: 0,09 µg/kg KG/min) notwendig.

Im weiteren Verlauf der Operation wurde die Norepinephrindosis bis maximal 0,2 µg/kg KG/min gesteigert, zusätzlich erhielt der Patient Vasopressin (bis 1 IE/h) sowie Milrinon (0,04 µg/kg KG/min). Unter dieser differenzierten Kreislauftherapie war die Hämodynamik anhaltend stabil.

Unter Einsatz von Alfentanil (Dosis: 26 µg/kg KG/h) und Propofol (Dosis: 0,25–1 mg/kg KG/h) ließ sich eine suffiziente Analgosedierung erreichen, eine adäquate Analgesie wurde zudem durch Lokalanästhetikaapplikation (Ropivacain 1 %) über den einliegenden PDK sichergestellt.

Aus chirurgischer Sicht gestaltete sich die offene onkologische Sigmaresektion komplikationslos, der Tumor konnte zügig und *in toto* reseziert werden (Schnitt-Naht-Zeit: 95 min, Blutverlust: 200 ml).

Postoperativ wurde der Patient zur weiteren Therapie und Überwachung auf die Intensivstation verlegt. Es persistierte eine Kreislaufinsuffizienz im Rahmen der bekannt eingeschränkten RV-Pumpfunktion über insgesamt 9 Tage, welche die Weiterführung der differenzierten Inotropika- und Vasopressorentherapie mit Milrinon, Norepinephrin und Vasopressin notwendig machte. Zur Senkung der RV-Nachlast wurden zudem Iloprost und Milrinon inhalativ verabreicht. Unter diesem Therapieregime gelang schließlich die hämodynamische Stabilisierung.

Postoperativ zeigte sich außerdem eine Verschlechterung der respiratorischen Insuffizienz Typ I, hier wurde neben intermittierender nicht-invasiver Beatmung mittels CPAP („continuous positive airway pressure“)/ASB („assisted spontaneous breathing“; Mund-Nasen-Maske) auch eine hoch dosierte Sauerstofftherapie in Form von HFNC eingesetzt. Innerhalb der folgenden 5 Tage gelang eine Reduktion der Sauerstoffgabe auf die vorbestehende Dosis. Der Patient konnte am 11. postoperativen Tag auf die viszeralchirurgische Allgemeinstation verlegt und von dort aus am 15. postoperativen Tag nach Hause entlassen werden.

#### Fallbericht II: Hemikolektomie bei Patientin mit PH

In diesem Fall berichten wir von einer 60-jährigen Patientin (160 cm, 54 kg) mit einer schweren präkapillären PH aufgrund einer Sarkoidose. Als Vorerkrankungen sind zusätzlich ein schweres allergisches Asthma bronchiale sowie rezidivierende Lungenarterienembolien bekannt. Die Patientin benötigt seit mehreren Jahren eine kontinuierliche Heimsauerstofftherapie mit 2 l/h. Initial stellte sich die Patientin aufgrund einer Verschlechterung des Allgemeinzustands und persistierender Dyspnoe im Sinne einer NYHA (New York Heart Association) III in der Inneren Medizin in unserem Krankenhaus vor. In der CT zeigten sich Hinweise auf einen Progress der PH, sodass weitere apparative Untersuchungen folgten (RHK, PET[Positronenemissionstomographie]-CT, Langzeit-EKG, TTE, LuFu). Die RHK-Untersuchung ergab einen zur Voruntersuchung stabilen Befund (PAPm: 54 mm Hg, Herzzeitvolumen: 7,5 l/min, CI [„cardiac index“]: 4,9 l/min/m^2^) bei mäßig aktiver kardialer Sarkoidose. Das Langzeit-EKG war normwertig, die TTE zeigte einen dilatierten RV (TAPSE 23) und eine erhaltene LVEF. Die LuFu ergab eine mäßige Obstruktion (VC_max_: 2,26 l, FEV_1_: 1,41 l) und eine schwere Diffusionsstörung (DLCOc SB [„single breath“]: 1,71 mmol/min/kPa, entsprechend 23 % vom Soll). Die kapilläre Blutgasanalyse unter Gabe von 2 l Sauerstoff bestätigte die respiratorische Insuffizienz (pO_2_: 66 mm Hg, pCO_2_: 48 mm Hg). In der PET-CT zeigte sich zusätzlich ein suspekter Untersuchungsbefund der rechten Kolonflexur. Die histopathologische Untersuchung einer endoskopisch gewonnenen Probe ergab ein neuroendokrines Karzinom, und es wurde die Indikation zur Hemikolektomie rechts gestellt. In der interdisziplinären Fallbesprechung wurde die Operation aufgrund der Vorerkrankungen in Regionalanästhesie in Form einer kombinierten Spinal- und Epiduralanästhesie (CSE) geplant. Präoperativ erfolgten ein Bridging der Marcumartherapie bei rezidivierenden Lungenarterienembolien und das zeitgerechte Absetzen vor der geplanten CSE. Vor Anlage der CSE wurde eine invasive arterielle Druckmessung (A. radialis links) etabliert und ein 3‑lumiger zentralvenöser Zugang (V. jugularis interna, 8,5 Fr) angelegt. Anschließend erfolgte die komplikationslose CSE (Höhe L 3/4) mit anschließender Anlage des Katheters. Für die Spinalanästhesie wurden Ropivacain 0,5 % sowie 5 µg Sufentanil und 7,5 µg Clonidin verwendet. Um eine ausreichende intraoperative Schmerzfreiheit im Bereich des Oberbauchs zu erreichen, wurde zusätzlich fraktioniert Ropivacain 1 % über den Periduralkatheter verabreicht. Bereits kurz nach Anlage der CSE war eine Noradrenalingabe von 0,09 µg/kg KG/min bei einsetzender Sympathikusblockade nötig. Auf Wunsch der Patientin erfolgte eine Analgosedierung mit Dexmedetomidin und Propofol. Zur intraoperativen Aufrechterhaltung des Kreislaufs war die Gabe von Noradrenalin 0,25 µg/kg KG/min und Vasopressin 2 IE/h nötig. Zudem erfolgte die Gabe von Milrinon 0,31 µg/kg KG/min. Es gelang eine zügige und komplikationslose Hemikolektomie rechts in offener Technik mit Anlage eines terminalen Stomas (Dauer: 61 min, Blutverlust: 100 ml). Postoperativ wurde die Patientin wach und unter anhaltend hoher Katecholaminpflichtigkeit auf die Intensivstation verlegt. Hier konnte die Katecholamintherapie nach rückläufiger Wirkung der Spinalanästhesie am 1. postoperativen Tag beendet werden. Der Gasaustausch zeigte sich unter der vorbekannten Sauerstofftherapie grenzwertig, sodass postoperativ eine intermittierende High-flow-Therapie nötig war. Eine adäquate Schmerztherapie wurde über eine PCA(„patient-controlled analgesia“)-Pumpe (Ropivacain 0,2 % und Sufentanil) durchgeführt. Die Patientin konnte am 2. postoperativen Tag kreislaufstabil und mit 2 l Sauerstoff über eine Nasenbrille auf die Allgemeinstation verlegt werden und nach weiteren 10 Tagen nach Hause entlassen werden.
